# Nanoarchitectured air-stable supported lipid bilayer incorporating sucrose–bicelle complex system

**DOI:** 10.1186/s40580-021-00292-5

**Published:** 2022-01-11

**Authors:** Hyunhyuk Tae, Soohyun Park, Gamaliel Junren Ma, Nam-Joon Cho

**Affiliations:** 1 School of Materials Science and Engineering, Nanyang Technological University, 50 Nanyang Avenue 639798, Singapore, Singapore; 2 China-Singapore International Joint Research Institute (CSIJRI), Guangzhou, 510000 China

**Keywords:** Supported lipid bilayer, Membranes, Bicelle, Lipids, Adsorption, Air stability, Sucrose, Mixtures

## Abstract

Cell-membrane-mimicking supported lipid bilayers (SLBs) provide an ultrathin, self-assembled layer that forms on solid supports and can exhibit antifouling, signaling, and transport properties among various possible functions. While recent material innovations have increased the number of practically useful SLB fabrication methods, typical SLB platforms only work in aqueous environments and are prone to fluidity loss and lipid-bilayer collapse upon air exposure, which limits industrial applicability. To address this issue, herein, we developed sucrose–bicelle complex system to fabricate air-stable SLBs that were laterally mobile upon rehydration. SLBs were fabricated from bicelles in the presence of up to 40 wt% sucrose, which was verified by quartz crystal microbalance-dissipation (QCM-D) and fluorescence recovery after photobleaching (FRAP) experiments. The sucrose fraction in the system was an important factor; while 40 wt% sucrose induced lipid aggregation and defects on SLBs after the dehydration–rehydration process, 20 wt% sucrose yielded SLBs that exhibited fully recovered lateral mobility after these processes. Taken together, these findings demonstrate that sucrose–bicelle complex system can facilitate one-step fabrication of air-stable SLBs that can be useful for a wide range of biointerfacial science applications.

**Supplementary Information:**

The online version contains supplementary material available at 10.1186/s40580-021-00292-5.

## Introduction

Supported lipid bilayers (SLBs) are nanoarchitectured, cell membrane-mimetic ultrathin (~ 5 nm) layers which are self-assembled on various types of hydrophilic surfaces [1–4]. SLBs’ outstanding lateral fluidity and compatibility with surface-sensitive analysis have demonstrated that they have great potential for use in a wide range of applications, such as analytical platforms in biology [5–10], diagnostic biosensors [11–14], and drug delivery systems [15–22]. To date, the most popular method to fabricate SLBs is the vesicle fusion, which is a bottom-up method involving the adsorption and spontaneous rupture of lipid vesicles on a solid surface [23–25]. This method is dependent on vesicle–substrate and vesicle–vesicle interactions, and thus vesicle adsorption and SLBs formation are affected by numerous experimental factors such as surface type, lipid composition, quality of vesicle, lipid concentration, and solution condition (e.g., osmotic pressure, solution pH, ion type, and ionic strength) [26–28]. This has spurred multitudinous efforts to develop new, simpler SLB fabrication strategies that are robust to experimental conditions [29]. One of the promising methods is the solvent-assisted lipid bilayers (SALB) method, which generates the SLBs on various solid supports based on the phase transition of phospholipids from inverted micelles and monomers in organic solvents to micelles and lamellar vesicles in aqueous buffer by solvent exchange [29–32]. Another useful method involves the adsorption of bicelles, which have a disk-like nanostructure composed of a mixture of long-chain and short-chain lipidic components [33, 34]. The advantages of this method are that it can be performed in various environmental conditions, does not require strict size control during sample preparation, and enables the efficient formation of SLBs from low concentrations of lipids [29, 35–38].

While the abovementioned and other methods have conquered many challenges to SLB fabrication, the restriction of SLB use to aqueous environments remains a major obstacle to their industrial applications, as SLBs exposed to air suffer fluidity loss and lipid-bilayer collapse [39, 40]. To address this obstacle, previous studies have fabricated air-stable and laterally mobile SLBs by immobilizing cholesterol on support [39], using zirconium phosphate [41], inserting copper phthalocyanine between bilayers [42], or crosslinking lipid molecules [43, 44]. Nevertheless, there is a need to develop effective fabrication methods that enable the formation of air-stable SLBs without requiring the modification of supports or lipid molecules, and/or the sophisticated control of parameters.

Studies have shown that sugar (i.e., sucrose or trehalose) molecules can be utilized for membrane stabilization [45, 46]. Sucrose, a disaccharide, has been demonstrated to protect cell membranes by regulating osmotic pressure, altering the phase-transition temperature of lipid molecules, and stabilizing biomolecular conformations during dehydration and freezing [47–50]. The stabilizing properties of sucrose molecules are due to their hydrogen-bonding interactions with the water molecules in the hydration layer and the multiple head groups of lipid molecules [51, 52]. Two mechanisms have been proposed for these interactions: the “water-replacement hypothesis” comprises direct interactions between sucrose molecules and lipids at the lipid interface [53–55], whereas the “hydration forces explanation” comprises indirect interactions between sucrose molecules and lipid bilayers expelled from the membrane surface [49, 51, 53].

**Scheme 1:**
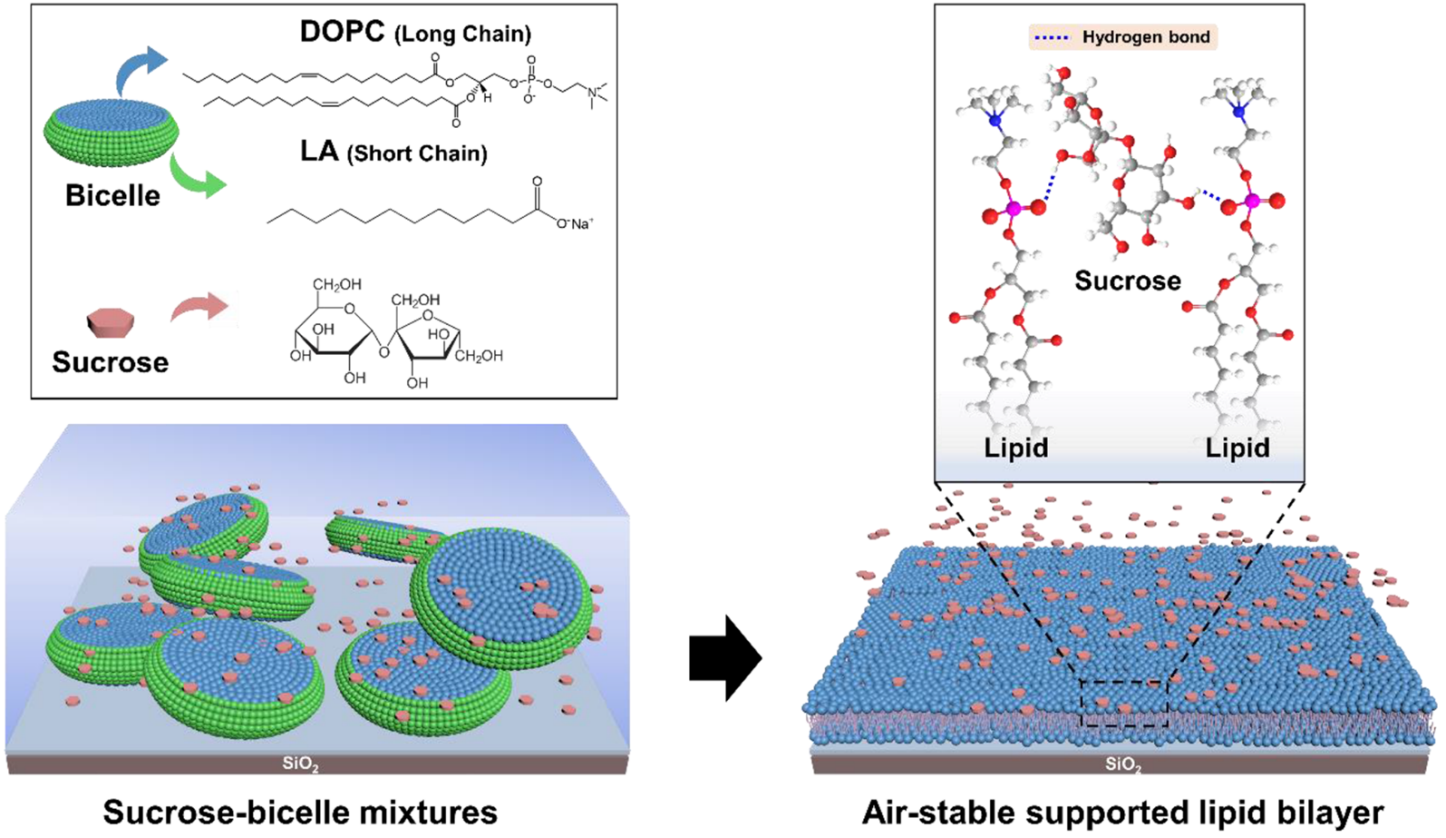
Rationale of air-stable SLBs formation from a sucrose–bicelle complex system. The potential of sucrose–bicelle mixtures to form SLBs was systematically investigated as a function of sucrose concentration by QCM-D, fluorescence microscopy, and FRAP techniques. The air-stable SLBs were fabricated by dehydrating the SLBs in sucrose solutions. The sucrose molecules in these air-stable SLBs interacted with phospholipids via hydrogen bonds, thereby protecting membranes from dehydration

Inspired by this membrane-protecting function of sucrose, we incorporated sucrose into a versatile bicelle adsorption strategy to fabricate air-stable SLBs (Scheme 1). Bicelle adsorption is a robust and versatile method to fabricate SLBs in different environmental conditions, and we extended this strategy in varying sucrose concentrations to develop sucrose–bicelle complex systems for SLBs formation. We first conducted quartz crystal microbalance with dissipation (QCM-D) monitoring and fluorescence microscopy imaging experiments to characterize bicelle adsorption and rupture behavior in sucrose solutions. We then performed fluorescence recovery after photobleaching (FRAP) experiments to evaluate the membrane fluidity of SLBs. Finally, we fabricated air-stable SLBs and measured their recovery of lateral lipid mobility after rehydration to identify the formation conditions that afforded SLBs with optimal properties, such as temporal air stability.

## Materials and methods

### Reagents

1,2-Dioleoyl-*sn*-glycerol-3-phosphocholine (DOPC) and 1,2-dioleoyl-*sn*-glycerol-3-phosphoethanolamine-*N*-(lissamine rhodamine B sulfonyl) (ammonium salt) (red; Rh–PE) lipids in chloroform were obtained from Avanti Polar Lipids (Alabaster, AL). Rh–PE has excitation/emission wavelengths of 560/583 nm. Lauric acid (LA) was obtained from Sigma-Aldrich (St. Louis, MO). The aqueous buffer used in all experiments was 10 mM *tris*(hydroxymethyl)aminomethane (Tris) buffer containing 150 mM NaCl (pH 7.5), prepared using Milli-Q water (Millipore Sigma, Burlington, MA). Sucrose was obtained from Affymetrix (Cleveland, OH), and sucrose solutions were prepared by dissolving defined concentrations of sucrose in Tris buffer.

### Preparation of sucrose–bicelle mixtures

First, DOPC lipids and LA in chloroform were added to a 4 mL glass vial. For fluorescence microscopy experiments, the DOPC lipids were doped with 0.5 mol% Rh–PE lipids. The solvent was then evaporated under a gentle flow of nitrogen gas and by subsequent incubation in a vacuum desiccator overnight at room temperature. Next, the dried DOPC/LA film (prepared above) was hydrated in an aqueous Tris buffer solution to a *q*-ratio of 2 (i.e., 1 mM DOPC lipids:0.5 mM LA). The resulting suspensions were then subjected to five freeze–thaw–vortex cycles, which involved the following steps: (1) submersion in liquid nitrogen for 1 min, (2) thawing in a 60 °C water bath for 5 min, and (3) vortexing for 30 s. Immediately before the experiment, an aliquot of the stock lipid suspension was diluted  ~ 32-fold using the sucrose solutions (from 0 to 50 wt% sucrose). The final DOPC and LA concentrations in all experiments were 0.031 mM and 0.0155 mM, respectively.

### Quartz crystal microbalance-dissipation (QCM-D)

QCM-D experiments were conducted using a Q-Sense E4 instrument (Biolin Scientific AB, Stockholm, Sweden). The quartz-crystal sensor chips had a fundamental frequency of 5 MHz, and the sensor surface had a 50-nm-thick sputter-coated silicon dioxide layer. Before the experiment, the sensor chips were successively rinsed with 1% (w/v) sodium lauryl sulfate (SDS) solution, deionized water, and 95% ethanol, dried under a flow of nitrogen gas, and then treated in an oxygen plasma chamber (PDC-002, Harrick Plasma, Ithaca, NY) for 1 min. The temperature of the QCM-D chambers was maintained at 25 °C. All solutions were added under continuous flow conditions using a peristaltic pump (Reglo Digital MS-4/6, Ismatec, Wertheim, Germany) at a flow rate of 50 µL/min. The Q-Soft software package (Biolin Scientific AB) was used to collect data at multiple odd overtones, and the data were reported at the 7th overtone and normalized according to the overtone number. Data processing was performed using the Q-Tools (Biolin Scientific AB) and OriginPro (OriginLab, Northampton, MA) software programs.

### Epifluorescence microscopy

Imaging experiments were conducted using a Nikon Eclipse Ti-E inverted microscope with a 20 ×  objective (NA 0.45) or a 60 ×  oil-immersion objective (NA 1.49). The excitation source was a mercury-fiber illuminator C-HGFIE Intensilight (Nikon, Tokyo, Japan), and the light was passed through a TRITC filter block (Ex 545/30, Em 605/70) for imaging red channels. An Andor iXon3 897 electron multiplying charge-coupled device (EMCCD) camera was used to record micrographs. Real-time bicelle adsorption experiments were conducted in home-built flow-through microfluidic chambers with glass coverslips (ibidi GmbH, Martinsried, Germany). The liquid sample was introduced at a flow rate of 50 μL/min via a peristaltic pump (Reglo Digital MS-4/6), and micrographs were recorded at a rate of 1 frame per 3 s. All measurements were conducted at room temperature (~ 25 °C).

### Fluorescence recovery after photobleaching (FRAP)

FRAP technique was used to measure the lateral diffusivity of fluorescently labeled Rh-PE lipids within SLBs [56]. A single-mode 523-nm laser source (100 mW, Coherent Inc., Santa Clara, CA) was used to photobleach a 20-μm diameter circular spot within the fabricated lipid membranes. Subsequently, fluorescence micrographs were captured using an Andor iXon3 897 EMCCD camera at 2-s intervals over 3–5 min to track the fluorescence recovery. Diffusivity was measured by the Hankel transform method to confirm the presence of bilayer domains [57]. The FRAP measurement was independently repeated a total of five times.

### Fabrication of air-stable SLBs

First, glass coverslips (ibidi GmbH) were successively rinsed with 1% w/v SDS solution, deionized water, and 95% ethanol, dried with nitrogen gas, and then treated in an oxygen plasma chamber (PDC-002) for 1 min. Next, the glass coverslips were incubated in sucrose–bicelle mixture of defined concentration for 30 min to form SLBs, and then washed with a sucrose solution of a defined concentration. The resulting sample-bearing coverslips were dried in air overnight or for 1 month, and finally rehydrated by dropwise addition of the buffer solution. Fluorescence imaging and FRAP analysis were performed to test the air stability of the rehydrated SLBs. All measurements were conducted at room temperature (~ 25 °C).

## Results and discussion

### Formation of supported lipid bilayers (SLBs)

**Fig. 1:**
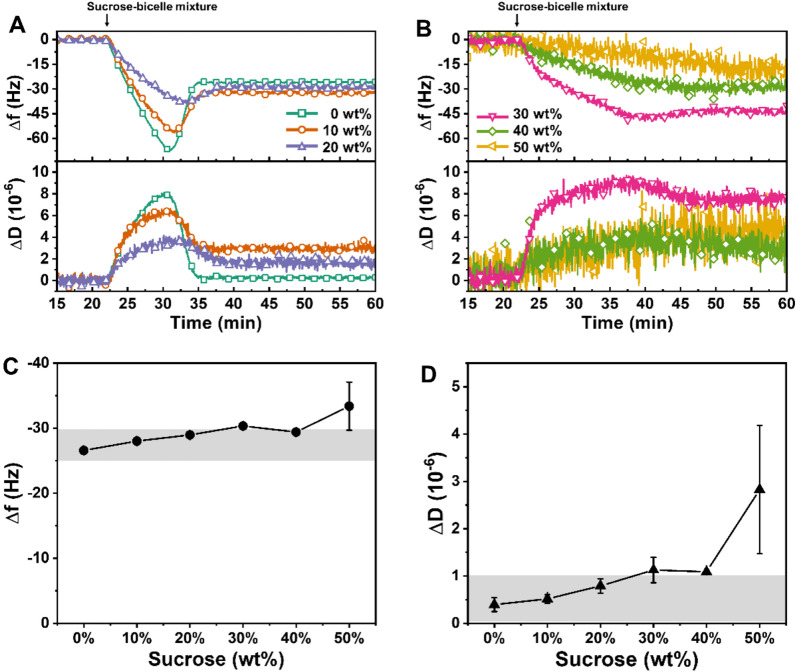
QCM–D characterization of bicelle adsorption onto silica surfaces in mixtures containing various concentrations of sucrose. Changes in Δ*f* and Δ*D* shifts were monitored as a function of time to track the bicelle adsorption. The normalized kinetic profiles of Δ*f* and Δ*D* shifts were determined by subtracting the respective Δ*f* and Δ*D* shifts of **A** 0–20 wt% and **B** 30–50 wt% sucrose solutions. The column graphs present the final values of **C** Δ*f* and **D** Δ*D* shifts after buffer washing (*n*  = 3, mean  ±  SD). The highlighted grey regions in the column graphs of final shifts denote typical values of a complete SLB

Bicelle adsorption is a well-established and versatile method for the formation of SLBs in various environments via the adsorption, fusion, and rupture of phospholipid bicelles onto silica surfaces [29, 36, 58, 59]. We extended this approach to form SLBs from sucrose–bicelles mixtures and performed QCM-D experiments to scrutinize the effect of sucrose on bicelles-mediated SLB formation. QCM-D measures the mass and viscoelastic properties of adsorbed layers by detecting shifts in resonance frequency (Δ*f*) and energy dissipation (Δ*D*), respectively. After the stabilization of a baseline signal in aqueous Tris buffer solution, we injected a sucrose solution (step 1, 7–22 min) followed by a sucrose–bicelles mixture (step 2, 22–62 min) to distinguish the effect of sucrose from that of bicelles adsorption in shifts (Additional file 1: Figure S1). The concentrations of bicelles were fixed at 0.031 mM DOPC and 0.0155 mM LA at a *q*-ratio of 2, based on the optimized condition [37]. The weakly adsorbed lipid and sucrose molecules were removed by washing with defined concentrations of sucrose solution (step 3, 62–77 min) and aqueous buffer solution (step 4, 77–85 min). In step 1, the Δ*f* shifts decreased by approximatel y − 46.8 ± 0.6 Hz to  − 660.7 ± 15.2 Hz, and the Δ*D* shifts increased by approximately 18.2 ± 0.3 × 10^−6^ to 267.2 ± 8.8 × 10^−6^, in proportion to the 10–50 wt% sucrose concentrations (Additional file 1: Figure S1B–F). To clearly characterize the kinetics of bicelles adsorption, the normalized kinetic profiles of Δ*f* and Δ*D* shifts were determined by subtracting the average values of sucrose solutions in step 1, and the final Δ*f* and Δ*D* shifts were presented together with these profiles in Fig. 1.

Figure 1A presents the normalized Δ*f* and Δ*D* shifts for bicelle adsorption in 0–20 wt% sucrose. Bicelle adsorption occurred via a two-step mechanism in all concentrations, which indicates that bicelles adsorbed onto a silica surface until a critical surface coverage was reached, then fused spontaneously to form SLBs [59]. Interestingly, the time span and the magnitude of Δ*f* and Δ*D* shifts to reach the critical surface coverage of adsorbed bicelles (Δ*f*_max_ and Δ*D*_max_, respectively) decreased by increasing sucrose concentrations. Generally, the frequency change after reaching its maximal value (i.e., critical surface coverage) is associated with the desorption of perpendicularly adsorbed bicelles and/or the loss of solvent captured within the bicellar mixtures [36]. Therefore, the trend of lower and slower adsorption of bicelles seemed to be attributed by the higher viscosity of sucrose solutions (see viscosity measurements in Additional file 1: Table S1) and concomitant interactions between sucrose and bicellar mixtures, inducing more horizontal yet slower attachment onto the surface by increasing the sucrose concentration. At higher concentration of sucrose (30–50 wt% sucrose), a similar trend was observed, signified by decreased attachment of bicellar mixtures (Fig. 1B). However, the two-step kinetics became obscured from 30 wt% sucrose incorporation, manifesting monotonic adsorption of bicelles rather than visible fusion and/or rupture signals. The result was also accompanied by higher magnitudes of noise fluctuation in the signal due to high contents of sucrose, requiring further assessment on the formation of SLB after rinsing with aqueous buffer.

After buffer washing, the final Δ*f* and Δ*D* shifts were measured to determine whether the adsorbed bicelles have formed SLBs (Fig. 1C, D). Although 10 and 20 wt% sucrose cases showed slightly higher final Δ*f* and Δ*D* shifts (− 28.0 to  − 29.0 Hz and 0.5–0.8 × 10^−6^_,_ respectively) compared to the 0 wt% control result, both values were within the typical range for complete SLBs, as indicated by grey shades in the figures. In 30 wt% sucrose, the final Δ*f* and Δ*D* shifts were  − 30.3 ± 0.4 Hz and 1.1 ± 0.3 × 10^−6^, and in 40 wt% sucrose, they were  − 29.4 ± 0.2 Hz and 1.1 ± 0.1 × 10^−6^, respectively. In all cases, these marginally higher values imply the association of sucrose with lipid molecules and a possible chance of the presence of intact or aggregated bicellar mixtures within SLBs in high sucrose concentrations. In contrast, 50 wt% sucrose condition yielded incomplete formation of SLB, demonstrated by large Δ*f* and Δ*D* shifts of  − 33.4 ± 3.7 Hz and 2.8 ± 1.4 × 10^−6^, respectively.

**Fig. 2:**
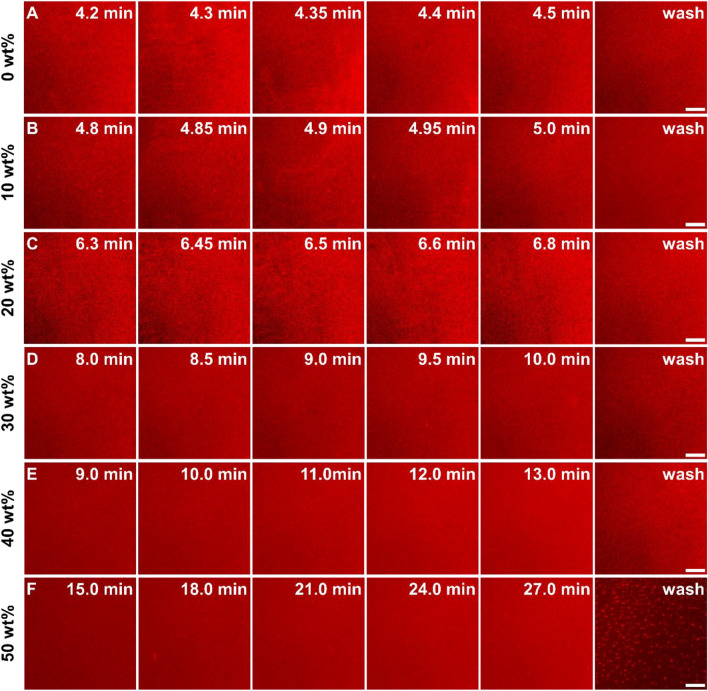
Time-lapse fluorescence micrographs of bicelle adsorption in various sucrose concentrations. For fluorescence microscopy imaging, bicelles were added onto a glass surface at *t*  = 0 min. The adsorption process in the following sucrose concentrations was then recorded: **A** 0 wt%, **B** 10 wt%, **C** 20 wt%, **D** 30 wt%, **E** 40 wt%, and **F** 50 wt%. All scale bars are 20 μm

To complement the QCM-D experiments, the real-time fluorescence microscopy imaging was also performed with fluorescently labeled lipid-doped within the long-chain lipid population (Fig. 2). At low concentrations of sucrose (0–20 wt%), the critical surface coverage and the subsequent fusion of bicelles were clearly captured within 4–7 min (Fig. 2A–C). This observation was consistent with the QCM-D results, showing two-step adsorption-fusion kinetics as well as the slower adsorption behavior with increasing sucrose concentrations. In contrast, there was negligible indication of bicelle fusion at 30–40 wt% sucrose case, although the uniform fluorescence coverage was observed after a buffer-washing step (Fig. 2D–E). Notably, the post-washing image did not show any sign of unruptured or aggregated bicellar mixtures, implying that the higher values obtained from the QCM-D measurements were likely due to the coupled sucrose with lipid molecules. In 50 wt% sucrose, the post-washing micrograph featured bright aggregates within dark adlayer, indicating the incomplete formation of SLB with the removal of the attached bicelles (Fig. 2F).

### Fluorescence recovery after photobleaching (FRAP)

**Fig. 3:**
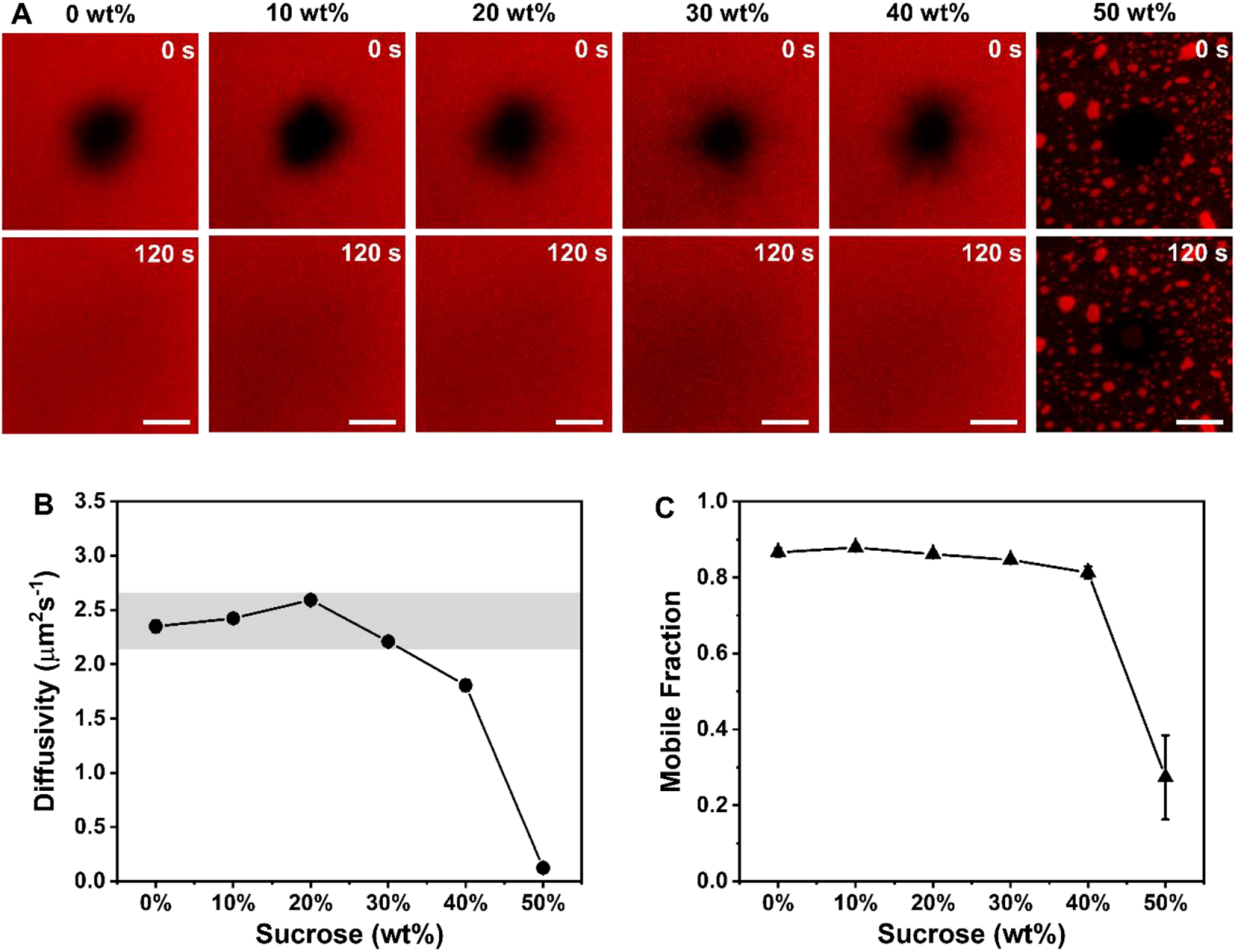
Fluorescence recovery after photobleaching (FRAP) analysis for the mobility characterization of lipid bilayers in various sucrose concentrations. **A** The fluorescence micrographs at 0 s and 120 s after photobleaching; fluorescence recovery within the photobleached region in 0–50 wt% sucrose. Scale bars are 20 μm. **B** Diffusion coefficient values and **C** mobile fractions for the lipid membranes formed from 0 to 50 wt% sucrose–bicelle mixtures (*n*  = 3, mean  ±  SD). The highlighted grey region in the graph of diffusivity values denotes typical values of a complete SLB

To further characterize the fluidity properties of the membrane, we conducted FRAP experiments to reveal the lateral diffusion of fluorescent lipid (Rh-PE) in the membrane by tracking the fluorescence recovery profile over time (Fig. 3). After the buffer washing step, 20 µm wide circular spot was photobleached in membrane, and the recovery of fluorescence was monitored. Up to 40 wt% sucrose, we found the near-complete recovery of photobleached spots within 2 min, confirming the high fluidity of fabricated SLBs in these conditions (Fig. 3A). However, there was insignificant fluorescence recovery in 50 wt% sucrose, highlighting the incomplete formation of SLB.

Intriguingly, SLBs formed in 10–30 wt% sucrose condition showed comparable diffusivity values to the control sample (0 wt% sucrose) (Fig. 3B), which demonstrate that they agree well with literature values reported for high-quality SLBs with the diffusivity of 2.2–2.6 μm^2^/s [37, 60, 61]. In addition, the SLBs from 10 to 30 wt% sucrose exhibited high mobile fractions of  > 85% (Fig. 3C). The high recovery profiles obtained from 10 to 30 wt% sucrose contrast with previously reported results, where  < 10 wt% sugar concentration showed the noticeably decreasing lateral mobility [62]. Our findings are likely attributed to the intrinsic structural difference between SLBs and giant unilamellar vesicles (GUVs), whereby the lipid diffusion in SLBs is more restricted than that in GUVs. For example, the lateral mobility of GUVs has been found to be  ~ two-fold higher than that of SLBs due to the interaction between lipids and the substrate [63]. The same study has reported almost identical diffusivity of SLBs in aqueous buffer and in low concentration of glucose solution [63]. Therefore, at low sucrose concentrations, it might be difficult to measure the effect of sucrose in altering the lateral mobility of SLBs.

On the other hand, 40 wt% sucrose showed a reduced diffusion coefficient of 1.8 μm^2^/s. To examine whether the slower lateral diffusion was attributed to the interaction between sucrose and lipid molecules, or to the presence of small fraction of unruptured bicellar mixtures within the membrane, we assessed the mobile fractions of SLBs (Fig. 3C). Strikingly, the mobile fraction of 40 wt% sucrose displayed 81%, which was comparable to those at 0–30 wt% sucrose. This observation suggests that the decreased mobility of lipids is primarily ascribed to the strong interaction between sucrose and lipid molecules rather than a minor population of unruptured bicellar mixtures. At the highest sucrose concentration (50 wt%), the diffusion coefficient was nearly 0 μm^2^/s as expected with  ~ 25% of mobile fraction. Together with QCM-D and fluorescence microscopy results, the different fraction of sucrose showed distinguishable characteristics in the formation of SLBs: complete SLB formation comparable to pure DOPC SLB occurred in 0–20 wt% sucrose via the two-step kinetics involving bicelle adsorption and fusion; SLB formation with pronounced sucrose interaction occurred in 30–40 wt% sucrose featured by monotonic kinetics with slight decrease in diffusion coefficient as well as mobile fraction; and unruptured bicelle adsorption occurred in 50 wt% sucrose.

### Characterization of the air stability of SLBs

Having formed SLBs from 10 to 40 wt% sucrose via the sucrose–bicelle complex system, we next determined the effect of sucrose concentrations on the protection of dehydrated SLBs. We selected 20 and 40 wt% sucrose concentrations to fabricate SLBs, then the samples were dehydrated overnight in air, followed by rehydration for the assessment of morphology and lipid mobility (details can be found in Sect. 2).

**Fig. 4:**
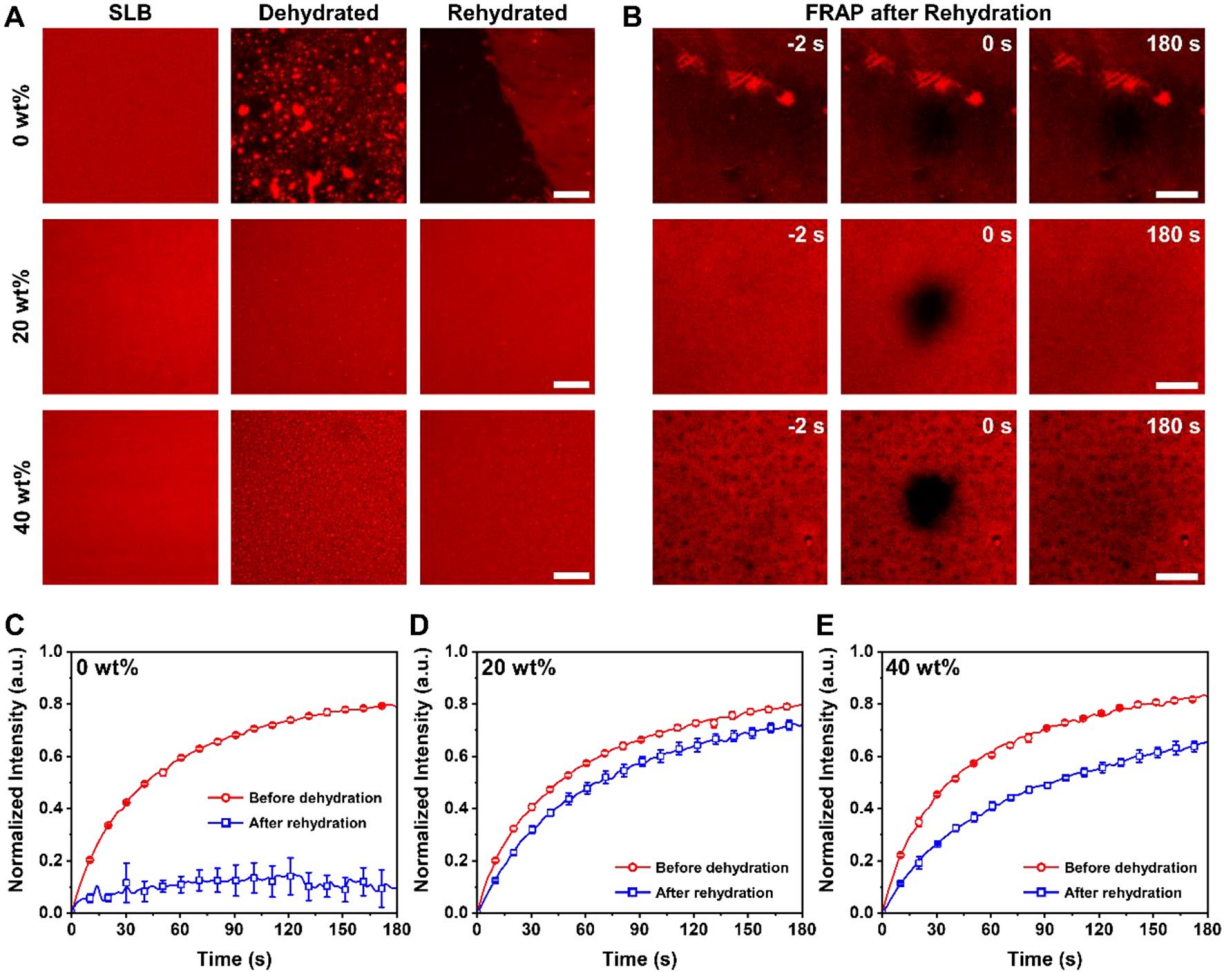
Air-stable SLB formation with sucrose–bicelle complex system. **A** Fluorescence microscopy images of SLBs formed in 0, 20, or 40 wt% sucrose (i.e., before dehydration), after dehydration, and after rehydration in a buffer. Scale bars are 50 μm. **B** FRAP results before and after photobleaching for lateral mobility characterization of lipids in rehydrated SLBs. Scale bars are 20 μm. Comparison of FRAP recovery curves for SLBs before dehydration and after rehydration, formed from **C** 0 wt%, **D** 20 wt%, and **E** 40 wt% sucrose

Upon dehydration (exposure to air) and rehydration cycle, the microscopic structure of SLBs formed without sucrose was severely damaged whereas SLBs formed from 20 and 40 wt% sucrose exhibited quite uniform fluorescence intensity, highlighting more stable structure by the incorporation of sucrose (Fig. 4A). As expected, the SLBs without sucrose lost its lateral diffusivity even after rehydration, which was confirmed by negligible fluorescence recovery of a photobleached spot (Fig. 4B, top) and fluorescence recovery profile in Fig. 4C. In contrast, 20 wt% sucrose demonstrated almost complete recovery after rehydration (Fig. 4B, middle) with  ~ 0.7 mobile fraction, which is only reduced by 8% compared to the freshly prepared SLBs (Fig. 4D). However, the SLBs formed from 40 wt% sucrose exhibited  ~ 0.6 mobile fraction, which is a quarter of reduction compared to the SLBs before dehydration (Fig. 4E).

Strikingly, highly fluorescent lipid aggregates were observed in dehydrated SLBs made from 20 to 40 wt% sucrose, albeit the number of bright specks in 20 wt% sucrose was much lower than in 40 wt% sucrose (Fig. 4A, note the different scales in Fig. 4A, B). Markedly, the aggregates in SLBs with 20 wt% sucrose disappeared after rehydration, whereas the majority of those bright aggregates remained in the rehydrated SLBs with 40 wt% sucrose. Moreover, after rinsing with buffer to conduct FRAP in a controlled environment, the defects were occurred on SLBs, manifested by immobile dark spots (rehydration, Fig. 4B, bottom). These results suggest that a significantly high concentration of sucrose might induce the local aggregation of phospholipids in SLBs during dehydration, thereby causing defects upon the rinsing step with aqueous buffer solution. Therefore, such lipid aggregate-generated defects seemed to cause the decrease in the lateral diffusion of rehydrated SLBs [64, 65].

In order to investigate the air stability of SLBs spanning longer duration of time, we also conducted FRAP analysis after one month of dehydration in air. After one month, the rehydrated SLBs formed from 20 wt% sucrose exhibited a similar lateral mobility to that of SLBs rehydrated after overnight (12 h) dehydration (Additional file 1: Figure S2A). In contrast, the SLBs formed from 40 wt% sucrose after one month of dehydration exhibited a severe reduction in the lateral mobility, as shown by almost a half reduction of mobile fraction compared to the freshly prepared SLBs formed in 40 wt% sucrose (Additional file 1: Figure S2B). Moreover, more noticeable and larger defects were found in the rehydrated SLBs after one month of dehydration, showing that the long-term storage in air of SLBs formed from high concentrations of sucrose might cause them to develop defects and subsequent decrease in their lateral mobility. Taken together, these results demonstrate that the air stability of SLBs is strongly dependent on the concentrations of sucrose, by affecting the membrane stabilization, local aggregation of lipids, and the number of defects in SLBs. Therefore, the prime air-stable SLB, in terms of maintaining the lipid mobility after rehydration, was fabricated by 20 wt% sucrose with bicellar mixtures while 40 wt% sucrose resulted in lipid aggregation and defects with subsequent reduction of lipid fluidity.

## Conclusions

In this work, we fabricated air-stable and fluid SLBs via sucrose–bicelle complex system that is inspired by the protective function of sucrose against cell membrane dehydration. Using a combination of QCM-D monitoring and fluorescence microscopy, we found that the sucrose–bicelle complex system was suitable to fabricate SLBs up to 40 wt% sucrose in a one-step process. The dependence on sucrose fraction demonstrates the importance of balancing strong interactions between sucrose molecules and lipid bilayers in order to facilitate high stability while maintaining SLB formation propensity. This air-stable SLB system can serve as an industrially useful platform for various biointerfacial science applications.

## Supplementary Information

**Additional file 1: Table S1.** Shear viscosity of sucrose solutions without/with bicelles, and bicelle sizes in sucrose–bicelle mixtures containing various concentrations of sucrose. **Figure S1.** QCM-D measurements of bicelle adsorption onto silica surfaces in various concentrations of sucrose (A–F, 0–50 wt% sucrose). The baseline of Δ*f* and Δ*D* shifts were recorded in Tris buffer containing 150 mM NaCl, and then measurements were made in the following solutions: (1) sucrose solution in Tris buffer; (2) sucrose–bicelle mixture for SLB formation; (3) sucrose solution in Tris buffer for washing step; (4) Tris buffer for final baseline to check the quality of SLB formation. **Figure S2. **Air stability of SLBs after 1 month of dehydration. (A) FRAP results and recovery curves for mobility characterization of SLBs made from 20 wt% sucrose and rehydrated after 1 month of dehydration. (B) Corresponding results for SLBs made from 40 wt% sucrose. All scale bars are 20 μm.

## Data Availability

The datasets used and/or analyzed during the current study are available from the corresponding author after submitting a reasonable request.

## Abbreviations

SLB: Supported lipid bilayer
QCM-D: Quartz crystal microbalance with dissipation
FRAP: Fluorescence recovery after photobleaching
DOPC: 1,2-Dioleoyl-*sn*-glycerol-3-phosphocholine
Rh-PE: 1,2-Dioleoyl-*sn*-glycerol-3-phosphoethanolamine-*N*-(lissamine rhodamine B sulfonyl) (ammonium salt)
LA: Lauric acid
Tris: *tris*(Hydroxymethyl)aminomethane
SDS: Sodium lauryl sulfate
EMCCD: Electron multiplying charge-coupled device
GUV: Giant unilamellar vesicle
