# Supplementary material for: Nanoarchitectured air-stable supported lipid bilayer incorporating sucrose–bicelle complex system
Source: Nano Converg. 2022 Jan 11;9:3. doi: 10.1186/s40580-021-00292-5 (PMC8752642; doi:10.1186/s40580-021-00292-5)
Supplement: Supplementary file 1 — Additional file 1: Table S1. Shear viscosity of sucrose solutions without/with bicelles, and bicelle sizes in sucrose–bicelle mixtures containing various concentrations of sucrose. Figure S1. QCM-D measurements of bicelle adsorption onto silica surfaces in various concentrations of sucrose (A–F, 0–50 wt% sucrose). The baseline of Δf and ΔD shifts were recorded in Tris buffer containing 150 mM NaCl, and then measurements were made in the following solutions: (1) sucrose solution in Tris buffer; (2) sucrose–bicelle mixture for SLB formation; (3) sucrose solution in Tris buffer for washing step; (4) Tris buffer for final baseline to check the quality of SLB formation. Figure S2. Air stability of SLBs after 1 month of dehydration. (A) FRAP results and recovery curves for mobility characterization of SLBs made from 20 wt% sucrose and rehydrated after 1 month of dehydration. (B) Corresponding results for SLBs made from 40 wt% sucrose. All scale bars are 20 μm. [file 40580_2021_292_MOESM1_ESM.docx]

*Supporting Information*

**Fabrication of air-stable supported lipid bilayer incorporating sucrose–bicelle complex system**

Hyunhyuk Tae,^†^ Soohyun Park,^†^ Gamaliel Junren Ma,^†^ and Nam–Joon Cho*^,†‡^

^†^School of Materials Science and Engineering, Nanyang Technological University, 50 Nanyang Avenue 639798, Singapore

^‡^China-Singapore International Joint Research Institute (CSIJRI), Guangzhou 510000, China

*Corresponding Author

**Email address:** njcho@ntu.edu.sg

**Investigation of shear viscosity and bicelle size distribution.** The effect of sucrose concentration on bicelle size distribution was investigated using a 90Plus particle size analyzer (Brookhaven Instruments Corporation, NY) at a scattering angle of 90° to minimize the reflection effect. Shear viscosity measurements of sucrose solutions and sucrose–bicelle mixtures were performed on a Modular Compact Rheometer (MCR) 501 (Anton Paar GmbH, Graz, Austria) with a 25-mm-diameter cone-plate geometry at a 2° angle and at room temperature. The shear viscosities of various sucrose concentrations were measured at a specific shear rate (355 s^−1^). All autocorrelation functions obtained were analyzed by the cumulant method and fitted to log-normal distribution(s) to obtain size distributions.

**Table S1.** Shear viscosity of sucrose solutions without/with bicelles, and bicelle sizes in sucrose–bicelle mixtures containing various concentrations of sucrose.

**
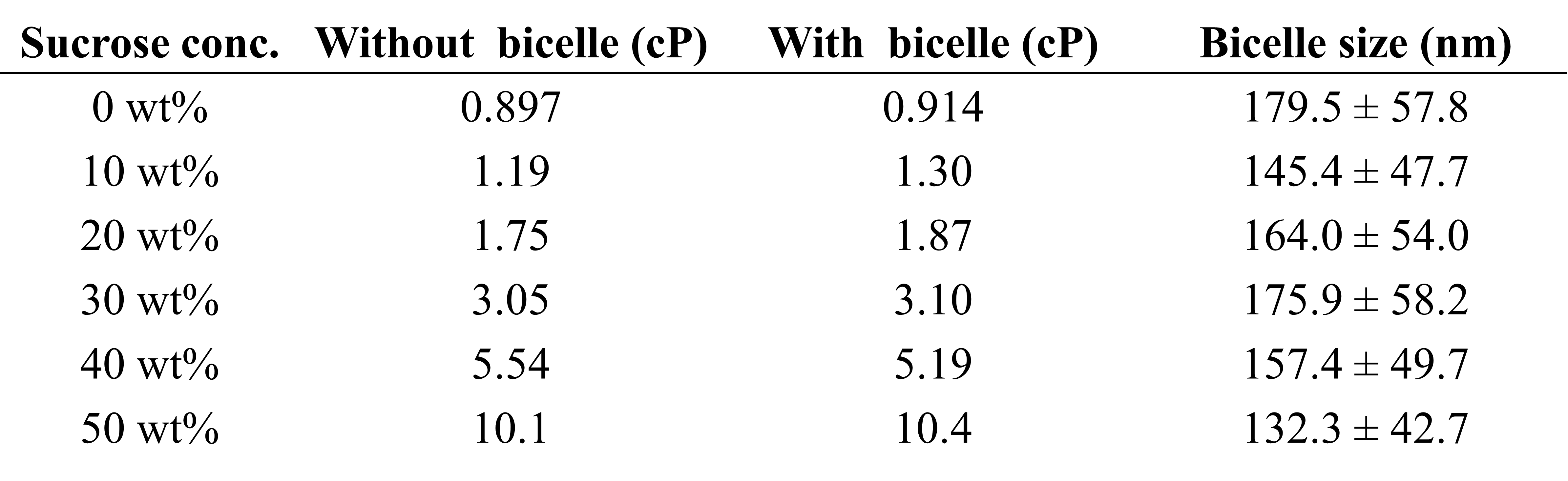
**

**
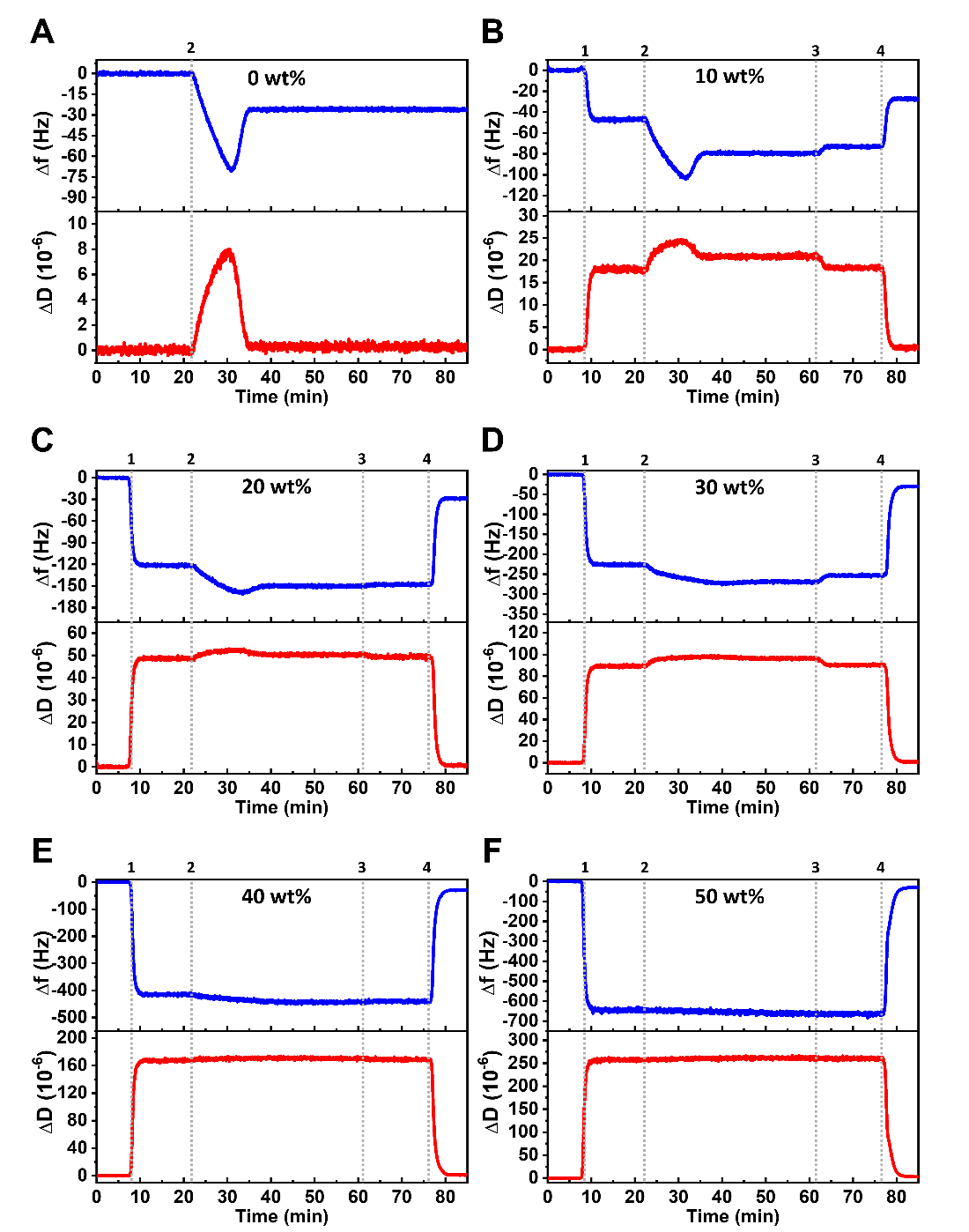
**

**Figure S1.** QCM-D measurements of bicelle adsorption onto silica surfaces in various concentrations of sucrose (A–F, 0–50 wt% sucrose). The baseline of Δ*f* and Δ*D* shifts were recorded in Tris buffer containing 150 mM NaCl, and then measurements were made in the following solutions: (1) sucrose solution in Tris buffer; (2) sucrose–bicelle mixture for SLB formation; (3) sucrose solution in Tris buffer for washing step; (4) Tris buffer for final baseline to check the quality of SLB formation.

**
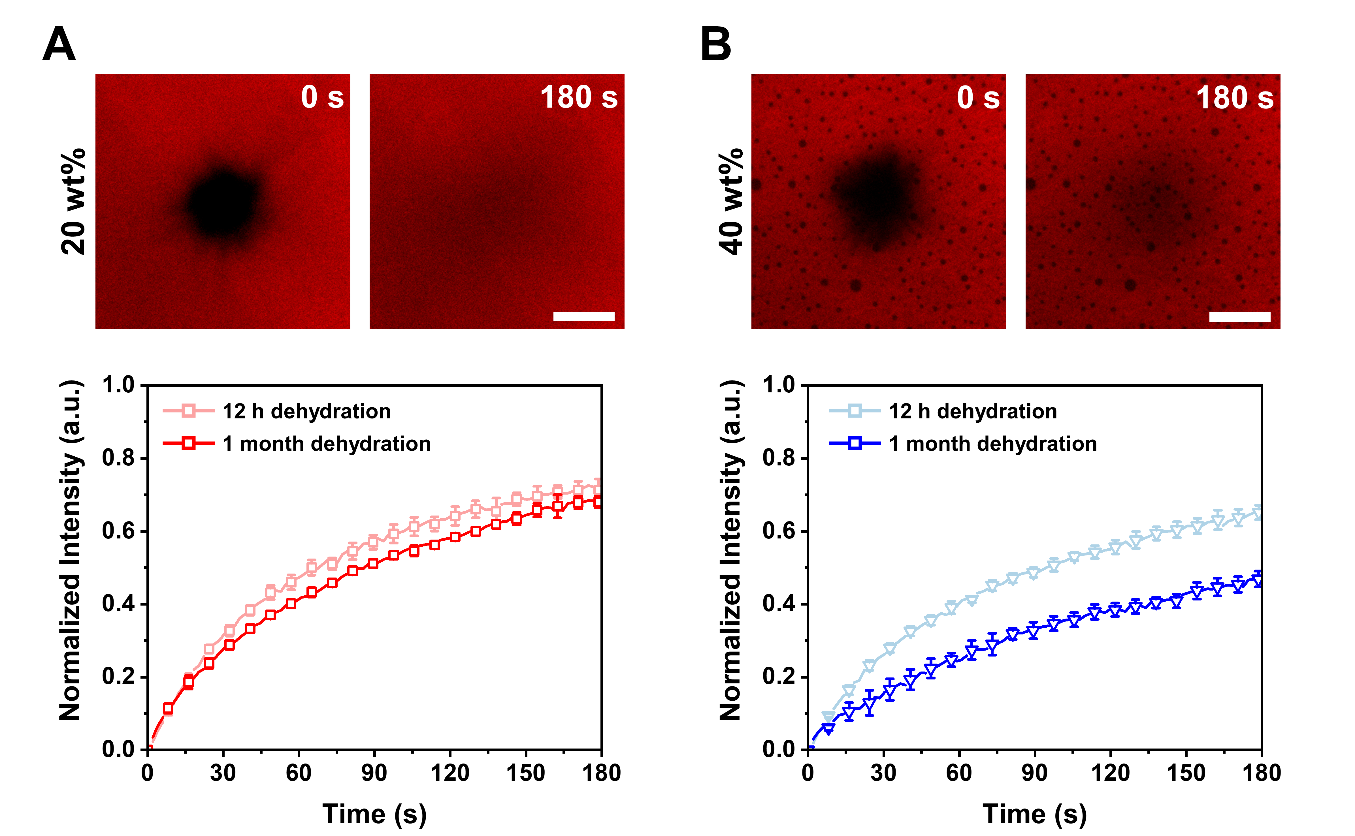
**

**Figure S2.** Air stability of SLBs after 1 month of dehydration. (A) FRAP results and recovery curves for mobility characterization of SLBs made from 20 wt% sucrose and rehydrated after 1 month of dehydration. (B) Corresponding results for SLBs made from 40 wt% sucrose. All scale bars are 20 μm.
